# Dissipation and Migration of Pyrethroids in *Auricularia polytricha* Mont. from Cultivation to Postharvest Processing and Dietary Risk

**DOI:** 10.3390/molecules23040791

**Published:** 2018-03-29

**Authors:** Jin-Jing Xiao, Jin-Sheng Duan, Yan-Can Wu, Yan-Hong Shi, Qing-Kui Fang, Min Liao, Ri-Mao Hua, Hai-Qun Cao

**Affiliations:** 1 School of Plant Protection, Anhui Agricultural University, Hefei 230036, Anhui, China; xiaojj187012@163.com (J.-J.X.); wuyancan1989@163.com (Y.-C.W.); shiyh@ahau.edu.cn (Y.-H.S.); qkfang@163.com (Q.-K.F.); liaomin3119@126.com (M.L.); 2 Provincial Key Laboratory for Agri-Food Safety, Anhui Agricultural University, Hefei 230036, Anhui, China; rimaohua@126.com; 3 Institute of Plant Protection and Agro-Product Safety, Anhui Academy of Agricultural Sciences, Hefei 230031, China; djszbzas@126.com; 4 Hefei Testing and Inspection Center for Agricultural Products Quality of Anhui Province, Hefei 230091, Anhui, China

**Keywords:** mushroom, pyrethroids, residue behavior, migration, processing factors

## Abstract

In order to ensure raw consumption safety the dissipation behavior, migration, postharvest processing, and dietary risk assessment of five pyrethroids in mushroom (*Auricularia polytricha* Mont.) cultivated under Chinese greenhouse-field conditions. Half-lives (*t*_1/2_) of pyrethroids in fruiting body and substrate samples were 3.10–5.26 and 17.46–40.06 d, respectively. Fenpropathrin dissipated rapidly in fruiting bodies (*t*_1/2_ 3.10 d); bifenthrin had the longest *t*_1/2_. At harvest, pyrethroid residues in *A. polytricha* (except fenpropathrin) were above the respective maximum residue limits (MRLs). Some migration of lambda-cyhalothrin was observed in the substrate-fruit body system. In postharvest-processing, sun-drying and soaking reduced pyrethroid residues by 25–83%. We therefore recommend that consumers soak these mushrooms in 0.5% NaHCO_3_ at 50 °C for 90 min. Pyrethroids exhibit a particularly low PF value of 0.08–0.13%, resulting in a negligible exposure risk upon mushroom consumption. This study provides guidance for the safe application of pyrethroids to edible fungi, and for the establishment of MRLs in mushrooms to reduce pesticide exposure in humans.

## 1. Introduction

With a delicate flavor and texture, as well as a high content of trace minerals, *Auricularia polytricha* (Mont.) Sacc., also known as wood ear, is one of the most popular edible mushrooms. It has a characteristic flavor and several known biological activities, including antioxidant, antitumor, anti-dementia, and hypocholesterolemic effects [1]. This has led to expansion of the global edible fungi market [2]. China in particular is the world’s largest producer and exporter of mushrooms, with an annual production output of around 50 million tons.

To meet increasing demand, the artificial cultivation of *A. polytricha* has expanded year on year, however, it is susceptible to a variety of insect infestations. The use of pesticides for combating insect pests and diseases in mushroom production has no doubt enhanced the production and quality of the products, but their indiscriminate use has led to the accumulation of pesticide residues, potentially harmful to humans and the environment [3,4]. In addition, sawdust is the main substrate for the cultivation of this mushroom, and is frequently reused, causing problematic residues in the substrate. Although not registered for this purpose in China, pyrethroids are used as insecticides for mushrooms by the majority of cultivators, causing exporting issues due to international restrictions. According to our survey, *A. polytricha* has been studied extensively for its artificial production, physiological properties, and nutritional value. However, little information is available on the behavior of pyrethroids in *A. polytricha* under cultivation conditions, especially in the migration and accumulation of pyrethroids in substrate-fruit body systems. Consequently, a thorough analysis of residue changes of pyrethroids in *A. polytricha* during cultivation is necessary.

The postharvest process, especially drying, is essential for preserving mushrooms, as fresh mushrooms have a high moisture content which causes perishablilty and microbial decomposition [5]. Numerous studies have demonstrated that food processing technology can contribute to pesticide dissipation [6,7]. The processing factor (PF) is the ratio of residue levels in processed products and their corresponding raw products, playing an important role in recommending maximum residue limits (MRLs) for processed products [8,9]. Moreover, MRLs are often only available for raw commodities. However, there are very few data concerning the behavior of pyrethroid residues in *A. polytricha* during postharvest processing. Accordingly, comprehensive knowledge of the fate of pyrethroids in the dry-storage-soak process is necessary to properly evaluate the human exposure from these pesticides.

The present work was designed to study the behavior of pyrethroids in mushrooms and their migration into the substrate-mushroom system. To investigate whether processing of the cultivated *A. polytricha* influences the levels of pyrethroids in the fruiting bodies, we also performed a series of experiments using various autoclave, storage, and drying conditions and soaking procedures. We also investigate the PFs during the postharvest dry-storage-soak process. This study may be helpful for establishing a rationale for pyrethroids MRLs in *A. polytricha* and provide relevant and useful data on the proper and safe use of pyrethroids.

## 2. Results and Discussion

### 2.1. Method Validation

Method validation was evaluated using selectivity, linearity, accuracy, and sensitivity. The selectivity was verified by comparison of the chromatograms obtained from fortified *A. polytricha* and substrates with those of blank samples. No interfering peaks were observed at the retention times of the tested analytes. Satisfactory linearity obtained with the squared correlation coefficients (*r*^2^) > 0.999 for the five pesticides analyzed by GC-ECD ranged from 0.005 to 1.0 mg·kg^−1^. Accuracy data were provided by recovery tests conducted in quintuplicate using the fortified blank samples at three fortification levels. Satisfactory accuracy was achieved with a recovery of 76.04–103.09% for dried *A. polytricha*, 81.68–96.39% for fresh *A. polytricha*, and 82.66–102.83% for the substrate (Table 1), respectively, which were within the ranges expected for residue analysis. Relative Standard Deviations (RSDs) < 10% for all pesticides demonstrated that the method is considered reliable enough for the routine analysis of the pesticide residues in this study. The sensitivity of the method was expressed in terms limits of detection (LODs), which were 0.50–1.60, 0.53–1.61, and 0.50–1.63 μg·kg^−1^ for dried *A. polytricha*, *A. polytricha*, and substrate, respectively. The LODs of the five pyrethroids, considered as the concentration that produced a signal-to-noise ratio of 10, ranged from 1.61 to 5.37 μg·kg^−1^ for all matrixes. This demonstrated that LODs and LOQs were also sufficiently reliable for detecting the likely concentrations of the incurred residues during processing.

### 2.2. Dissipation of Pyrethroid Residues in Substrate and Fruiting Bodies

**Figure 1:**
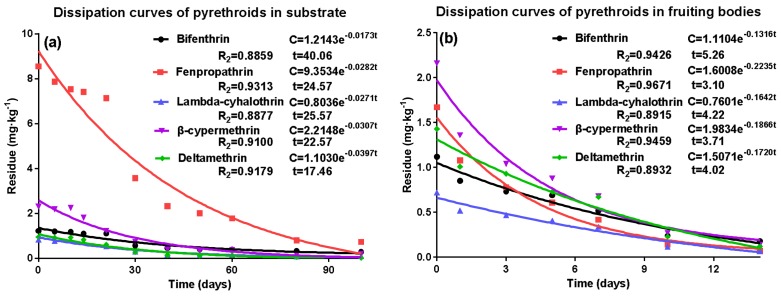
The dissipation curve of the five pyrethroid residues in the substrate (**a**) and fruiting body of *A. polytricha* (**b**). Percentage digestion represent the percentage reduction of residues. Results are reported as mean ± standard error (S.D.) (calculated from three independent experiments).

Kinetic dissipation was carried out to investigate if the substrate could pollute the environment or future crops. The dissipation curves of pyrethroids in the substrate after sterilization are shown in Figure 1a. The degradation trends of pyrethroids in the substrate followed first order kinetics, and their initial mean deposits ranged from 1.60–13.70 mg·kg^−1^ (Table S1) with half-lives (*t*_1/2_) of 17.46–40.06 d. Fan et al. (2013) [10] reported that the half-lives of five pyrethroids in a growing medium of pleurotus ostreatus were 25.48–54.59 d. Variability in degradation rate was observed among different types of samples, further proof that the crop characteristics affect the persistence of the pesticide. A sharp decrease was observed for the majority of pyrethroid residues 21 d after application, except for β-cypermethrin (14 d). At the end of the sampling period (100 d after application), more than 85% of residues in pyrethroids in all testing sites had been degraded, except for bifenthrin (77.05%). Bifenthrin may exhibit a longer half-life due to the absorption intensity of sawdust [11]. From the above, it is proposed that pyrethroids are fairly persistent in the substrate, and this warrants further study.

At the applied dosage of 1.5 times the recommended dosage, the initial residues in fruiting bodies were 1.12 mg·kg^−1^ for bifenthrin, 1.67 mg·kg^−1^ for fenpropathrin, 0.72 mg·kg^−1^ for lambda-cyhalothrin, 2.16 mg·kg^−1^ β-cypermethrin, and 1.43 mg·kg^−1^ for deltamethrin (Table S2). After 10 d, 80% of pyrethroids had degraded, and fenpropathrin and lambda-cyhalothrin levels in fruiting bodies were less than 0.07 mg·kg^−1^ 14 d after application. The degradation trends of pyrethroids in fruiting bodies also followed first order kinetics (Figure 1b). The *t*_1/2_ values of pyrethroids in fruiting bodies ranged from 3.10–5.26 d, demonstrating a faster degradation than that in the substrate.

Several factors, including environmental factors (light, moisture, and pH), the crop characteristics, and physical and chemical properties of the pesticide (water solubility and volatility), have played a fundamental role in affecting pesticide persistence in crops [12]. *A. polytricha* was mainly cultivated in greenhouse. In this paper, the differences in *t*_1/2_ could be raised by growth dilution factor and the type of plants, but less so by environmental factors.

### 2.3. Terminal Residues of Pyrethroids in A. polytricha

The terminal residue levels of five pyrethroids in *A. polytricha* could be detected after the last application at the recommended dose and the 1.5 times dose (Table 2). The results indicated that at the lower dosage of pyrethroids, the terminal residues ranged from 0.06–0.94 mg·kg^−1^ in *A. polytricha*. At the high dosage, the five pyrethroid residues ranged from 0.11–1.59 mg·kg^−1^, suggesting that the dosage played an important role on the residue. The harvest intervals after application also affected the residue of pyrethroids in *A. polytricha*. As harvest intervals increased, the residues declined considerably, for example, over 50% reduction of β-cypermethrin was observed in that collected after 3–5 d of application at the high dosage. It is expected that the highest residues are found at the shortest pre-harvest intervals. The residues of the five pyrethroids in *A. polytricha* also increased with an increase in application times, but the differences in the residues were not significant at the two experimental sites. 

Regardless of the dosage or spray times, 7 d after spraying, the final residues of pyrethroids in all of the *A. polytricha* mushroom samples (except fenpropathrin which was sprayed once, collecting after 7 d of application at a lower dosage) were above the respective MRL (0.05 mg·kg^−1^ of bifenthrin and β-cypermethrin, 0.1 mg·kg^−1^ of fenpropathrin, 0.02 mg·kg^−1^ of lambda-cyhalothrin, and 0.01 mg·kg^−1^ of deltamethrin) established in the EU, Japan, or China (Table 2). Human exposure to pyrethroids may cause subacute or chronic toxicity like neurotoxicity, immunotoxicity, reproductive and developmental toxicity [13,14]. This study shows that significant pesticide residue was detected in *A. polytricha* under the designed experiment, outside the acceptable residual level limits. It is thus evident that the residues of pyrethroids in fresh *A. polytricha* pose a potential health risk and this should be an area of continued focus. Considering the high terminal residue in *A. polytricha*, the dosage of application and harvest interval should be controlled in order to make it safer, and this needs further research. 

### 2.4. Migration of Pyrethroids in the Substrate-Mushroom System

Substrate for cultivation of *A. polytricha* was prepared using straw, sawdust and cottonseed hull. These ingredients allow the migration of pyrethroids into the fruiting body during its growth process, and expose the edible fungi to potential pesticide residues. In this paper, the migration of five pyrethroids into *A. polytricha* fruiting bodies from substrate was investigated over a 15-d period (Table S3).

Lambda-cyhalothrin was the only pesticide standard found in the fruiting bodies to show moderate recovery. The highest migration efficiency of lambda-cyhalothrin into fruiting bodies was observed in the first harvest (95 d), followed by a notable drop, which reflects the saturation points of the matrices. Migration into fruiting bodies was significantly affected by the initial concentration of the lambda-cyhalothrin, similar to previous findings in the paddy by Barik et al. [15]. The sawdust may be derived from polluted tree and substrate is frequently reused. However, this behavior means that the pyrethroids have a weak migration capacity, which improves the safety and quality of *A. polytricha*. Considering the accumulation of lambda-cyhalothrin in fruiting bodies, which was over 0.02 mg·kg^−1^, we do not recommend the use of lambda-cyhalothrin in mushrooms. This is the first time that lambda-cyhalothrin has been shown to migrate into edible fungi.

### 2.5. Effects of Processing

#### 2.5.1. Effects of Autoclaving on the Residue Dissipation Behaviors

**Figure 2:**
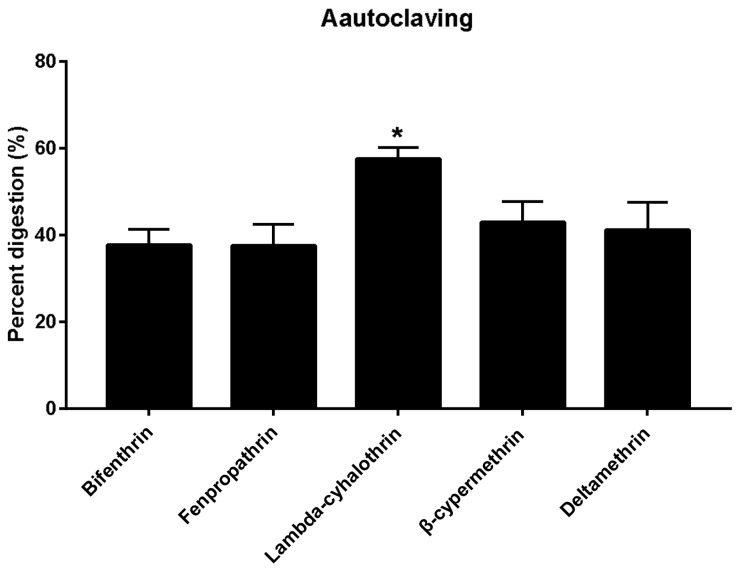
Effects of autoclaving process on residue levels of the five pyrethroids in *A. polytricha.* Results are reported as mean ± standard error (S.D.) (calculated from three independent experiments). Percentage digestion represent the percentage reduction of residues. The asterisks indicate significant differences in the percentage digestion among the five pyrethroids or different parameters in processed *A. polytricha*. (* *p* value < 0.05).

The substrate used in *A. polytricha* cultivation is frequently sterilized using high-pressure-steam before use. Moist heat can accelerate the decomposition of pyrethroids, and the dissipation was beyond 20% [16,17]. In order to determine the effect of autoclaving on the removal of pyrethroids in the substrate, fortified samples were prepared for pesticide analysis prior to, and immediately after, sterilization in an autoclave (Figure 2). Significant differences between results prior to, and after, sterilization was observed after autoclaving. Furthermore, our survey demonstrated that autoclaving significantly increased the levels of pyrethroids, with a range of 37.59–57.65%, consistent with that found in the earlier work [16]. The water involved in autoclaving could entrain pesticide molecules causing co-distillation, while heat promotes the degradation and evaporation of pesticides during the process [18]. Variations in degradation may be due to the chemical nature of the individual pesticides, including vapor pressure, water solubility, volatility, and tendency to bind to a matrix. Hence, it can be concluded that lambda-cyhalothrin is, in contrast to other pesticides, considerably more unstable during autoclaving.

#### 2.5.2. Effects of Two Drying Methods on the Residue Dissipation Behaviors

**Figure 3:**
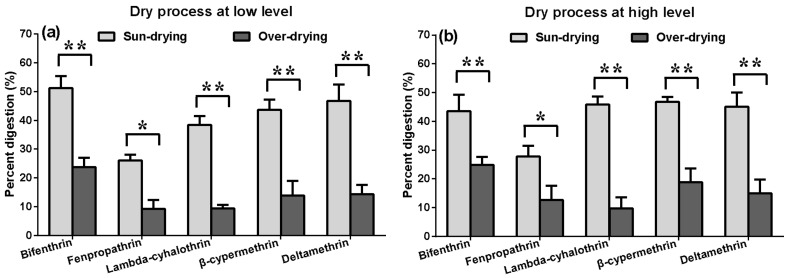
Effects of sun- and oven-drying at low (**a**) and high level (**b**). Results are reported as mean ± standard error (S.D.) (calculated from three independent experiments). Percentage digestion represent the percentage reduction of residues. The asterisks indicate significant differences in the percentage digestion among the five pyrethroids or different parameters in processed *A. polytricha*. (* *p* value < 0.05 and ** *p* value < 0.01).

Fresh *A. polytricha* contain porphyrins, which are potentially hazardous to consumers [19]. The drying process can significantly decrease the levels of porphyrins. Thus, consumers usually purchase the cultivated mushroom as a processed product. We investigated the influence of the two drying methods, sun- and oven-drying, on the residues of the pyrethroids at the low (Figure 3a) and high (Figure 3b) level. The results showed that the two drying methods have an effect on the degradation of the five insecticides used on *A. polytricha*. The percentage digestion of the pyrethroids ranged from 9.30–51.25%, with bifenthrin and deltamethrin showing the most degradation, followed by β-cypermethrin, whereas fenpropathrin and lambda-cyhalothrin showed comparatively less. Compared to oven-drying, sun-drying played a more significant role in promoting the degradation of the five pesticides, e.g., the amount of bifenthrin digestion was 43.61–51.25% and 23.76–24.82% for sun-drying and oven-drying treatments, respectively. In addition, the percentage digestion of the pyrethroids changed, but not statistically significantly, between the high- and low-dosage treatments after processing. These results suggest that in addition to a higher temperature, photolysis may also be a decisive factor that contributes to pesticide degradation during the drying process [20,21]. Interestingly, the drying process caused evaporation of water from products (around 70%), whereas the concentration of pesticides in final samples increased 2–3 times. A similar phenomenon has also been observed in other products, e.g., chili peppers [22], suggesting that the moisture content should be considered during the drying. At present, the MRL of edible fungi accepted in China does not distinguish between the fresh or dried products, so it is necessary to further subdivide.

#### 2.5.3. Effects of Storage on the Residue Dissipation Behaviors

**Figure 4:**
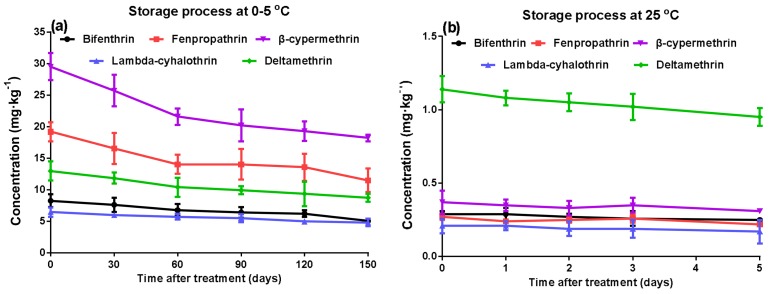
Effects of storage of fresh *A. polytricha* at low temperature (0–5 °C) (**a**) and dried *A. polytricha* at room temperature (25 °C) (**b**). Results are reported as mean ± standard error (S.D.) (calculated from three independent experiments).

When bought fresh and stored for a shorter time, mushrooms are usually kept in the refrigerator. To store for longer times, mushrooms are either frozen or dried. We followed the percentage digestion of the pyrethroids during storage at low temperature (0–5 °C, Figure 4a) and room temperature (25 °C, Figure 4b). Our survey showed that the percentage digestion of the pyrethroids was related to the storage time. For fresh *A. polytricha*, a small percentage digestion (around 15–20%) after 5 d treatment was observed during the low-temperature storage. However, no significant differences were observed with increasing time. For room-temperature storage, the mean loss of pyrethroids was 32–40% after 150 d treatment, which indicates that the degradation of pyrethroids is slow at room temperature, especially from 60–120 d. At the same time, the percentage digestion at room-temperature storage was slower than that at low-temperature storage. The result is in contrast to the previous studies with other pesticide residues in agricultural commodities. This be justified by storing *A. polytricha* in a dry, air-tight container, in a cool dark place [23].

#### 2.5.4. Effects of Soaking on the Residue Dissipation Behaviors

**Figure 5:**
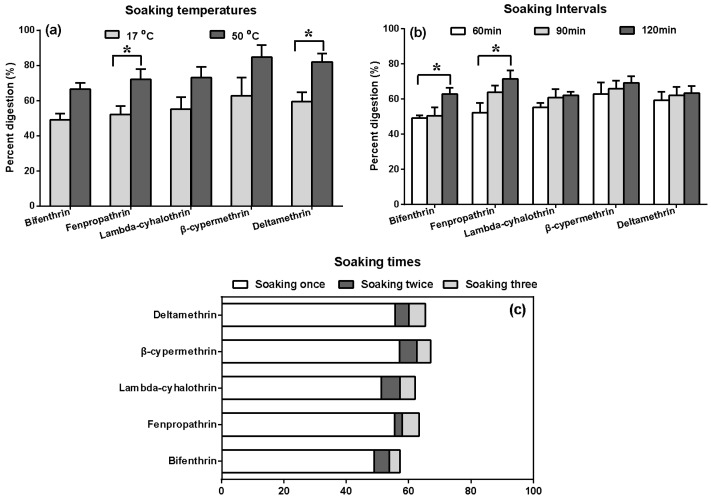
Effects of soaking temperatures (**a**), soaking intervals (**b**), and soaking times (**c**). Results are reported as mean ± standard error (S.D.) (calculated from three independent experiments). Percentage digestion represent the percentage reduction of residues. The asterisks indicate significant differences in the percentage digestion among the five pyrethroids or different parameters in processed *A. polytricha*. (* *p* value < 0.05).

Soaking with water is an essential before household cooking, to wash and to make the *A. polytricha* absorb enough water, to restore the dried mushrooms to a similar state as fresh ones. As shown in Figure 5a–c, soaking had a significant effect on the reduction of pyrethroids, with a mean 60% decrease. The loss increases with increasing temperature (Figure 5a), intervals (Figure 5b), and times (Figure 5c). The average removal percentage of the three methods showed no statistically significant differences, whereas a moderate difference was observed between pesticides. Bifenthrin showed a comparatively lower loss of the residues during the various soaking procedures. 

The differences in results might be related to the different physico-chemical properties of the tested pyrethroids, including water-octanol partition coefficients (Log K_ow_) and water solubility [24,25]. Moreover, washing has been found to reduce pesticide residues that are loosely attached to the surface [26]. The skin of *A. polytricha* is mainly pectin. Pyrethroids had no effects on the systemic properties [27], but were mainly present on the surface of the fruiting bodies. The percentage removal of these pesticides through soaking indicates that soaking is an effective way to remove these four pesticides.

**Figure 6:**
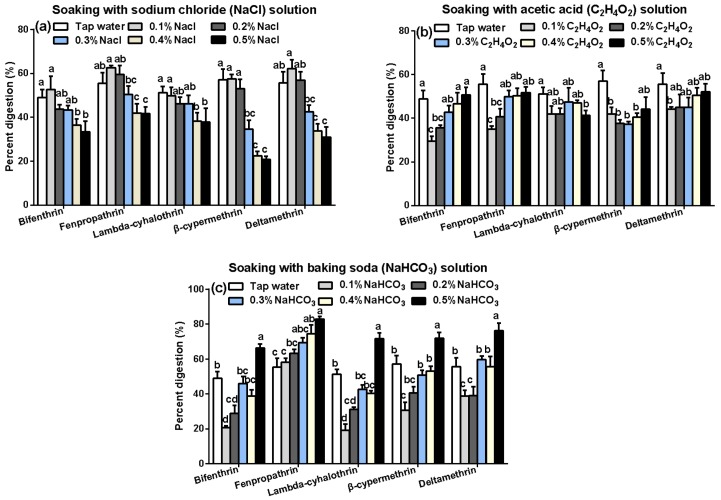
Effects of sodium chloride (NaCl) (**a**), acetic acid (C_2_H_4_O_2_) (**b**), and sodium bicarbonate (NaHCO_3_) (**c**) solutions on the removal percent of the five pyrethroid residues in *A. polytricha* during household-soaking. Percentage digestion represent the percentage reduction of residues. Different minor case letters at the top of the columns indicate significant differences at a *p* value of 0.05.

In a further report, pesticide residues and removal percentage were affected with various solutions, e.g., sodium chloride (NaCl), acetic acid (C_2_H_4_O_2_), or sodium bicarbonate (NaHCO_3_) [28]. As shown in Figure 6, after soaking with different solutions, the mean losses of bifenthrin, fenpropathrin, lambda-cyhalothrin, β-cypermethrin, and deltamethrin were 20.73–66.25%, 35.06–82.98%, 19.08–71.57%, 20.73–71.86%, 30.91–76.34%, respectively. The most efficient removal from *A. polytricha* was observed using 0.5% NaHCO_3_, while soaking with NaCl and C_2_H_4_O_2_ were less efficient in comparison. With a few exceptions, the residue of the five pesticides decreased by increasing the concentration of NaCl (Figure 6a), in contrast to the results with C_2_H_4_O_2_ (Figure 6b) and NaHCO_3_ (Figure 6c). This tendency might be related to the saturation of the pyrethroids, with NaCl enhancing the saturation, and thus causing lower transfer to the infusion. The average removal percentage of pyrethroids by the NaCl solution is lower than that of tap water, except at the 0.1% concentration, whereas removal of pyrethroids by NaHCO_3_ was more effective than tap water at high concentrations, especially for fenpropathrin. This may be due to the alkaline nature of the solution (pyrethroid pesticides are stable in acidic and neutral conditions but aqueous hydrolysis occurs under alkaline conditions) can enhance the solubility, and the release from the *A. polytricha* [29]. A similar process was studied by Wang et al. [30] who found that soaking in a dilute alkaline solution (pH 9.0) only eliminated 37.82% of the residues of the β-cypermethrin in contaminated vegetables, further demonstrating that pesticides have different behavior in different materials.

The results suggested that sun-drying and soaking were very effective procedures for reducing pesticide residues in the fruiting bodies. Moreover, we recommend processing the mushroom with sun-drying and soaking at 50 °C for 90 min with 0.5% NaHCO_3_ (pH 8.35), as suggested by the present findings.

### 2.6. Processing Factors and Dietary Risk

We know that different food processing technologies will lead to different residue levels, and the disposition of the residues in various processed products [31]. The PFs lay an important role in performing a risk assessment for a pesticide under a specific treatment in specific commodity [9,32]. The calculated PFs for the five insecticides after processing are shown in Table 3. Results show that all PFs were <1, indicating that their residual ratios are decreased during the entire process. During the soaking process, the PFs of the five pesticides were all less than 0.34, indicating that this process considerably reduced their residues in the *A. polytricha*. Moreover, the PFs of the overall process for bifenthrin, fenpropathrin, lambda-cyhalothrin, β-cypermethrin, and deltamethrin were 0.10, 0.08, 0.13, 0.10, and 0.09, respectively, demonstrating that the whole process can significantly reduce the residue of pesticides in *A. polytricha* postharvest processing.

## 3. Materials and Methods

### 3.1. Chemicals and Samples

*A. polytricha* and substrate were purchased from a local market as the main materials. None of the studied pyrethroids were detected in the samples. Pyrethroid analytical standards (bifenthrin (98.0%), fenpropathrin (99.2%), lambda-cyhalothrin (99.2%), β-cypermethrin (99.2%), and deltamethrin (98.4%)) were purchased from the National Pesticide Quality Supervision and Inspection Center (Beijing, China). Standard stock solutions of the pyrethroids were prepared in *n*-hexane. Subsequently, matrix-matched standard solutions were prepared by adding the appropriate stock solution using blank extracts of *A. polytricha* and substrate samples. All solutions were stored at 4 °C until use. The substances obtained through commercial sources included 25 g·L^−1^ bifenthrin emulsifiable concentrate (EC) (Jiangsu Yangnong Chemical Group Co., Ltd., Yangzhou, Jiangsu, China), 200 g·L^−1^ fenpropathrin EC (Tianjin Xingguang Chemical Co., Ltd., Tianjin, China), 25 g·L^−1^ lambda-cyhalothrin EC (Zhejiang Shijia Technology Co. Ltd., Huzhou, Zhejiang, China), 25 g·L^−1^ β-cypermethrin EC (Qingdao Han Sheng Bio Polytron Technologies Inc., Qingdao, China), and 25 g·L^−1^ deltamethrin EC (Jiangsu Yangnong Chemical Group Co., Ltd.).

### 3.2. Cultivation Conditions, Pest Surveys, and Field Trials

Trials were conducted in cultures of the commercial strains 120 of *A. polytricha* in mushroom growing rooms using a plastic bag cultivation method [33]. The greenhouse had three trials, 90 cubic meters (m^3^) in each trial. Of which, two trials for the field dissipation behavior study were carried out by different application treatments (premixing and spraying) during the cultivation of *A. polytricha*, and one trial was conducted for the study of the processing process.

Before cultivation, each polyethylene bag (height 38 cm, diameter 10 cm) was filled with 2.5 kg of substrate (74% of sawdust, 25% of cottonseed hull, 1–2.5% of lime) and sterilized at 100 °C for 14–16 h. After cooling, the substrate was inoculated with 9 cm^2^ mycelial agar discs. The spawn was incubated at 25 °C under dark conditions until the substrate was fully colonized. Then, a temperature of 25 ± 3 °C and a relative humidity of 65–75% were maintained throughout cultivation. 

#### 3.2.1. Pesticides for Trials of Premixing Treatment

Three separate field trials were conducted in an experimental greenhouse according to the guidelines on pesticide residue trials. Multi-mixing different pesticides (1:750 (*w*/*w*, single dosage) and 1:500 (*w*/*w*, 1.5 times dosage) for the ratio of the pesticide to the substrate, to ensure an ample amount of pesticide residues) was premixed with the non-sterilized or sterilized substrates before inoculation of the *A. polytricha*. The substrates without addition of any pesticide were used as control. A total of 12 substrates were used for each treatment. The prepared substrates premixed with pesticides were sealed into polyethylene bags and then the *A. polytricha* were inoculated to the substrates and cultivated conventionallyas mentioned previously. Approximately 200.0 g of non-sterilized and sterilized substrate (single dosage) was harvested at 0, 5, 10, 14, 21, 30, 40, 50, 60, 80, and 100 d to analyze the effect of autoclaving on the removal of pyrethroids in substrate and their dissipation behavior. Another 1.0 g of sterilized substrates diluted to a single and 1.5 times dosage were harvested at the first (95 d), second (110 d), and third harvest (125 d) to investigate the migration of pyrethroids in the substrate-mushroom system. The 12 samples of the same batch were taken and their content was mixed and then separated into triplicate for analysis.

#### 3.2.2. Pesticides for Trials of Spraying Treatment

The spraying treatment was designed as residue dynamic trials, consisting of 6 treatment plots and one control plot without application of the pesticide. Each plot consisted of an area of 10 m^2^ (approximately 12 of polyethylene bag). The recommended dosage (0.83 ai. g/m^2^) and 1.5 times the recommended dosage (1.25 ai. g/m^2^) used in vegetables was applied. The pesticides were diluted with 0.25 L of water and sprayed in each plot (10 m^2^) when the fruiting bodies of *A. polytricha* had grown to maturity (average height of 2 cm). Subsequently, about 200.0 g of *A. polytricha* were picked at 0 (2 h), 1, 3, 5, 7, 10, 14 d after spraying. 

To study the terminal residue, another two dosage levels, 0.83 ai. g/m^2^ (recommended dosage) and 1.25 ai. g/m^2^ was sprayed in another two treatment plots. Each dosage application was sprayed once or twice with an interval of 7 d. Subsamples were collected at pre-harvest intervals of 3, 5, and 7 d after the last spray. Samples of the same batch were taken and their content was mixed and then separated into triplicate for analysis.

### 3.3. Postharvest Processing and Home-Soaking Experiments

In general, the processing procedures of *A. polytricha* include three stages; storage, drying, and soaking. In this study, pyrethroids were applied at 0.83 and 4.15 ai. g/m^2^ (5 times the recommended dosage) to ensure an ample amount of pesticide residues. Then, subsamples were collected at 2 h to determine the variation of pesticide residue during the processing procedure. The detailed processing procedures are as follows.

Drying: for sun-drying, 1.0 kg of the fortified fresh samples were spread over a 1 m^2^ of area (open air, 35–40 °C) until samples were fully dry. For oven-drying, the samples were placed in an oven at 50 °C for 6 h (until the weight no longer changed).

Storage: fresh *A. polytricha* stored at 0–5 °C in the refrigerator (Shanghai Kendall cold storage company, Shanghai, China) for 0, 1, 2, 3, and 5 d; dried samples stored at room temperature in a dry environment for 1, 30, 60, 90, 120, and 150 d.

Soaking: to investigate the effect of water temperature, 100 g of dried *A. polytricha* was soaked in water (2 L) at either 17 °C or 50 °C for 60 min. The effect of soaking intervals was examined at intervals of 60, 90, and 120 min in the same manner. To investigate the effect of soaking time, 100 g of sample was immersed in 2 L of water at room temperature for 10 min. Subsequently, the liquid portion was decanted and collected as the first soaking. Then, another 2 L of water was poured into the original utensil to soak the residual samples and to obtain a second soaking. A third infusion was also obtained by the same process. In addition, samples were immersed in water (2 L) with varying levels (0.1%, 0.2%, 0.3%, 0.4%, and 0.5%) of baking soda, vinegar, and salt at room temperature for 90 min.

### 3.4. Gas Chromatography (GC) Analysis

The spiked substrate (5.0 g) or fresh or dried mushroom (10.0 g) was exactly weighed into a 50 mL centrifuge tube, and extracted and cleaned according to our previously reported method [34]. The elute was concentrated to dryness and reconstituted in 5.0 mL of *n*-hexane for GC with electron capture detection (ECD).

A 2010 Plus Network GC system (Shimadzu, Kyoto, Japan) equipped with a ^63^Ni ECD and a Rtx-5 capillary column (30 m × 25 μm × 2.5 μm film thickness) was used for pesticide analysis. The detector and injection temperatures were maintained at 300 °C and 250 °C, respectively. The GC settings were as follows: the initial oven temperature was held at 60 °C for 1 min, ramped at 30 °C/min to 180 °C and 5 °C/min to 250 °C for 5 min, and then ramped at 3 °C/min to 280 °C for 1 min. The carrier gas was nitrogen (99.999%) at a flow rate of 1.5 mL·min^−1^, and the sample injection volume was 2 μL in the splitless mode.

### 3.5. Statistical Analysis

Data are expressed as the mean ± S.D. Statistical analysis for each parameter was performed using analysis of variance (ANOVA) followed by Tukey’s test [35]. All figures were drawn using the software Origin Pro 9.0 (Origin Lab Corporation, Northampton, MA, USA). Differences among means were considered statistically significant at a *p* value of 0.05. The PFs are calculated and considered by the Joint FAO/WHO Meeting on Pesticide Residues (JMPR) [36]:

(1) $$\mathrm{PF}=\frac{\mathrm{The}\;\mathrm{residue}\;\mathrm{levels}\;\mathrm{of}\;\mathrm{processed}\;\mathrm{commodities}}{\mathrm{The}\;\mathrm{residue}\;\mathrm{levels}\;\mathrm{of}\;\mathrm{the}\;\mathrm{raw}\;\mathrm{commodities}}$$

PF values < 1 (=reduction factor) indicate a decrease of the residue in the processed commodity, while values > 1 (=concentration factor) indicate a concentration effect of the processing steps [37].

## 4. Conclusions

In this study, changes in bifenthrin, fenpropathrin, lambda-cyhalothrin, β-cypermethrin, and deltamethrin residue levels from cultivation to postharvest processing (storage and drying) and home soaking were studied. Many factors such as the chemical properties of the particular pesticide, processing procedure, etc. could affect the removal of pesticide residues. The soaking process was found to be the most effective for the reduction of pesticide residues. The present study provides residue data which may be useful for establishing MRL and assessing the amount of pyrethroid residues in mushrooms under Chinese field conditions. Moreover, the present findings suggest that a need for implementation of these safety intervals before harvesting and marketing such edible fungi.

## Figures and Tables

**Table 1:** Recoveries (%) and relative standard deviations (RSDs) for the five pyrethroids in *A. polytricha* and substrate samples at three spiking levels (*n* = 5).

Pesticides	Fortified Level (mg·kg^−1^)	Dried Mushroom			Fresh Mushroom			Fortified Level (mg·kg^−1^)	Substrate		
		Average Recovery (%) ^a^	RSD (%) ^b^	LQD/LOQ (μg·kg^−1^)	Average Recovery (%)	RSD (%)	LQD/LOQ (μg·kg^−1^)		Average Recovery (%)	RSD (%)	LQD/LOQ (μg·kg^−1^)
Bifenthrin	0.005	82.51 ± 3.65 ^c^	3.99	0.91/2.93	95.02 ± 5.74	2.82	0.86/2.87	0.01	82.66 ± 4.01	2.58	0.85/2.44
	0.05	76.89 ± 2.54	1.76		90.42 ± 4.52	3.64		0.1	93.93 ± 4.15	4.33	
	0.5	83.56 ± 4.12	0.49		81.68 ± 3.25	2.30		1	88.52 ± 3.87	2.76	
Fenpropathrin	0.005	103.09 ± 5.31	2.16	0.75/2.61	94.71 ± 3.34	3.89	0.79/2.64	0.01	101.02 ± 3.65	1.67	0.66/2.55
	0.05	80.44 ± 4.33	2.78		90.60 ± 4.26	4.35		0.1	93.22 ± 3.77	2.00	
	0.5	82.66 ± 3.87	0.95		85.30 ± 3.39	3.45		1	87.93 ± 2.96	2.19	
Lambda-cyhalothrin	0.005	80.12 ± 3.26	1.72	0.50/1.62	85.96 ± 3.46	2.92	0.53/1.77	0.01	101.01 ± 3.82	2.49	0.50/1.61
	0.05	76.04 ± 2.57	1.01		93.24 ± 4.55	3.73		0.1	96.74 ± 3.54	3.73	
	0.5	90.13 ± 4.57	1.71		96.39 ± 4.12	1.87		1	87.07 ± 2.99	2.21	
β-cypermethrin	0.005	98.63 ± 5.44	3.71	1.60/5.34	84.73 ± 1.41	4.30	1.61/5.34	0.01	102.83 ± 5.54	2.87	1.63/5.37
	0.05	98.13 ± 4.75	2.31		87.16 ± 3.25	3.26		0.1	94.22 ± 4.93	1.11	
	0.5	82.67 ± 3.52	0.61		89.61 ± 5.75	2.35		1	87.07 ± 4.43	2.45	
Deltamethrin	0.005	93.57 ± 5.11	2.26	1.39/4.68	90.49 ± 3.76	3.62	1.42/4.73	0.01	101.47 ± 5.86	4.99	1.30/4.11
	0.05	81.96 ± 3.57	2.70		86.52 ± 3.13	1.93		0.1	96.53 ± 4.37	3.63	
	0.5	80.13 ± 2.54	0.87		92.08 ± 4.84	5.36		1	84.88 ± 2.58	2.55	

^a^ Five replicate extractions were performed for each treatment; ^b^ Relative standard deviation for reproducibility in %; ^c^ Standard deviation.

**Table 2:** Final residues of the five pyrethroids in *A. polytricha* (*n* = 3).

Pesticides	Application Dosage (ai. ^a^ g/m^2^)	Spray Times	Days after Application (Mean ± SD ^b^)		
			3	5	7
Bifenthrin	1.25	1	0.49 ± 0.06	0.21 ± 0.01	0.11 ± 0.02
		2	0.56 ± 0.02	0.29 ± 0.04	0.21 ± 0.01
	0.83 ^c^	1	0.64 ± 0.10	0.22 ± 0.03	0.16 ± 0.02
		2	077 ± 0.05	0.34 ± 0.05	0.28 ± 0.03
Fenpropathrin	1.25	1	0.71 ± 0.04	0.33 ± 0.02	0.09 ± 0.04
		2	0.79 ± 0.05	0.42 ± 0.08	0.21 ± 0.01
	0.83	1	0.96 ± 0.12	0.28 ± 0.01	0.17 ± 0.02
		2	1.12 ± 0.09	0.51 ± 0.06	0.37 ± 0.01
Lambda-cyhalothrin	1.25	1	0.39 ± 0.02	0.14 ± 0.03	0.06 ± 0.02
		2	0.44 ± 0.04	0.19 ± 0.06	0.11 ± 0.01
	0.83	1	0.53 ± 0.05	0.14 ± 0.02	0.11 ± 0.05
		2	0.63 ± 0.06	0.24 ± 0.01	0.16 ± 0.03
β-cypermethrin	1.25	1	0.94 ± 0.02	0.37 ± 0.03	0.15 ± 0.01
		2	1.06 ± 0.01	0.51 ± 0.05	0.27 ± 0.01
	0.83	1	1.29 ± 0.17	0.35 ± 0.04	0.22 ± 0.02
		2	1.59 ± 0.06	0.67 ± 0.04	0.33 ± 0.04
Deltamethrin	1.25	1	0.84 ± 0.10	0.30 ± 0.05	0.13 ± 0.03
		2	0.96 ± 0.02	0.43 ± 0.02	0.25 ± 0.01
	0.83	1	1.14 ± 0.03	0.31 ± 0.04	0.23 ± 0.04
		2	1.34 ± 0.10	0.56 ± 0.01	0.31 ± 0.06

^a^ ai., active ingredient; ^b^ Standard deviation; ^c^ Not registered in China for use on edible fungi. The recommended dosages of these compounds referred to pesticides for use in other plants.

**Table 3:** Processing factors (PFs) for the five pyrethroids after different processes (*n* = 3).

Process	Bifenthrin	Fenpropathrin	Lambda-Cyhalothrin	β-Cypermethrin	Deltamethrin
Drying	0.49	0.74	0.62	0.56	0.53
Storage	0.61	0.60	0.75	0.62	0.67
Soaking	0.34	0.17	0.28	0.28	0.24
Overall process	0.10	0.08	0.13	0.10	0.09

## Supplementary Materials

The following are available online, Table S1: Pyrethroid residues in the substrate at different time intervals (*n* = 3), Table S2: Pyrethroid residues in fruiting bodies at different time intervals (*n* = 3), Table S3: Migration and accumulation of pyrethroids in the substrate-mushroom system (*n* = 3).
