# Supplementary material for: Dissipation and Migration of Pyrethroids in Auricularia polytricha Mont. from Cultivation to Postharvest Processing and Dietary Risk
Source: Molecules. 2018 Mar 29;23(4):791. doi: 10.3390/molecules23040791 (PMC6017079; doi:10.3390/molecules23040791)
Supplement: Supplementary file 1 [file molecules-23-00791-s001.pdf]

Table S1. Pyrethroids residues in substrate at different time intervals ($n = 3$)

Interval time (day)	Bifenthrin		Fenpropathrin		Lambda-cyhalothrin		Beta-cypermethrin		Deltamethrin	
	Residues ($mg \cdot kg^{-1} \pm$ SD [a])	Dissipation rate [b] (%)	Residues ($mg \cdot kg^{-1} \pm$ SD)	Dissipation rate (%)	Residues ($mg \cdot kg^{-1} \pm$ SD)	Dissipation rate (%)	Residues ($mg \cdot kg^{-1} \pm$ SD)	Dissipation rate (%)	Residues ($mg \cdot kg^{-1} \pm$ SD)	Dissipation rate (%)
0	1.22 ± 0.11	/	8.55 ± 0.84	/	0.83 ± 0.07	/	2.3 ± 0.12	/	0.94 ± 0.10	
5	1.19 ± 0.15	2.46	7.87 ± 0.51	7.95	0.77 ± 0.12	7.23	2.18 ± 0.25	5.22	0.91 ± 0.10	3.19
10	1.17 ± 0.24	4.10	7.53 ± 0.65	11.93	0.81 ± 0.08	3.61	2.24 ± 0.34	2.61	0.92 ± 0.09	2.13
14	1.1 ± 0.08	9.84	7.41 ± 0.48	13.33	0.72 ± 0.11	13.25	1.80 ± 0.21	21.74	0.82 ± 0.11	12.77
21	1.1 ± 0.13	9.84	7.13 ± 0.39	16.61	0.54 ± 0.10	34.94	1.22 ± 0.10	46.96	0.61 ± 0.05	35.11
30	0.56 ± 0.07	54.10	3.57 ± 0.25	58.25	0.30 ± 0.09	63.86	0.73 ± 0.13	68.26	0.27 ± 0.04	71.28
40	0.45 ± 0.09	63.11	2.32 ± 0.28	72.87	0.15 ± 0.04	81.93	0.39 ± 0.08	83.04	0.14 ± 0.02	85.11
50	0.4 ± 0.05	67.21	2.01 ± 0.19	76.49	0.13 ± 0.05	84.34	0.31 ± 0.04	86.52	0.11 ± 0.06	88.30
60	0.38 ± 0.07	68.85	1.77 ± 0.21	79.30	0.16 ± 0.01	80.72	0.36 ± 0.06	84.35	0.13 ± 0.02	86.17
80	0.32 ± 0.05	73.77	0.79 ± 0.10	90.76	0.07 ± 0.01	91.57	0.15 ± 0.04	93.48	0.06 ± 0.01	93.62
100	0.28 ± 0.06	77.05	0.72 ± 0.11	91.58	0.10 ± 0.03	87.95	0.18 ± 0.02	92.17	0.02 ± 0.01	97.87

[a] Standard deviation. [b] Percentage degradation after spraying.

Table S2. Pyrethroids residues in fruiting bodies at different time intervals (n = 3).

Interval time (day)	Bifenthrin		Fenpropathrin		Lambda-cyhalothrin		Beta-cypermethrin		Deltamethrin	
	Residues (mg·kg^{-1} ± SD [a])	Dissipation rate (%)	Residues (mg·kg^{-1} ± SD)	Dissipation rate (%)	Residues (mg·kg^{-1} ± SD)	Dissipation rate (%)	Residues (mg·kg^{-1} ± SD)	Dissipation rate (%)	Residues (mg·kg^{-1} ± SD)	Dissipation rate (%)
0	1.12 ± 0.21	/	1.67 ± 0.34	/	0.72 ± 0.10	/	2.16 ± 0.41	/	1.43 ± 0.35	/
1	0.85 ± 0.07	24.11	1.08 ± 0.10	35.33	0.52 ± 0.09	27.78	1.36 ± 0.25	37.04	1.01 ± 0.16	29.37
3	0.73 ± 0.10	34.82	0.78 ± 0.05	53.29	0.47 ± 0.03	34.72	1.04 ± 0.20	51.85	0.93 ± 0.15	34.96
5	0.69 ± 0.08	38.39	0.61 ± 0.06	63.47	0.41 ± 0.09	43.06	0.88 ± 0.12	59.26	0.73 ± 0.11	48.95
7	0.52 ± 0.03	53.57	0.42 ± 0.05	74.85	0.34 ± 0.08	52.78	0.68 ± 0.09	68.52	0.67 ± 0.08	53.15
10	0.24 ± 0.05	78.57	0.14 ± 0.06	91.62	0.12 ± 0.03	83.33	0.27 ± 0.05	87.5	0.23 ± 0.04	83.92
14	0.18 ± 0.07	83.93	0.07 ± 0.02	95.81	0.07 ± 0.02	90.28	0.14 ± 0.03	93.52	0.12 ± 0.06	91.61

[a] Standard deviation.

Table S3. Migration and accumulation of pyrethroids in substrate-mushroom system (n = 3)

Interval time (day)	Multiple dilution	Residue Levels (mg·kg⁻¹)									
		Bifenthrin		Fenpropathrin		Lambda-cyhalothrin		Beta-cypermethrin		Fenvalerate	
		Substrate	Fruiting body	Substrate	Fruiting body	Substrate	Fruiting body	Substrate	Fruiting body	Substrate	Fruiting body
The first harvest (95 day)	Single dosage [a]	0.12 ± 0.02 [b]	ND [c]	0.95 ± 0.06	ND	0.10 ± 0.01	0.09 ± 0.02	0.82 ± 0.01	ND	0.09 ± 0.01	ND
	1.5 times dosage	0.40 ± 0.05	ND	1.27 ± 0.01	ND	0.18 ± 0.03	0.16 ± 0.03	1.45 ± 0.05	ND	0.16 ± 0.02	ND
The second harvest (110 day)	Single dosage	0.06 ± 0.01	ND	0.45 ± 0.04	ND	0.06 ± 0.01	0.03 ± 0.01	0.45 ± 0.06	ND	0.03 ± 0.01	ND
	1.5 times dosage	0.26 ± 0.04	ND	0.75 ± 0.02	ND	0.10 ± 0.02	0.09 ± 0.01	0.88 ± 0.02	ND	0.09 ± 0.01	ND
The third harvest (125 day)	Single dosage	0.04 ± 0.01	ND	0.34 ± 0.03	ND	0.04 ± 0.01	0.01 ± 0.001	0.43 ± 0.03	ND	0.01 ± 0.003	ND
	1.5 times dosage	0.15 ± 0.02	ND	0.81 ± 0.08	ND	0.09 ± 0.01	0.02 ± 0.004	0.79 ± 0.02	ND	0.02 ± 0.005	ND

[a] Single dosage, 1:750 (*w*/*w*) for the ratio of the pesticide to the substrate. [b] Standard deviation. [c] *ND,* not detected.
